# Supplementary material for: Network analyses identify liver‐specific targets for treating liver diseases
Source: Mol Syst Biol. 2017 Aug 21;13(8):938. doi: 10.15252/msb.20177703 (PMC5572395; doi:10.15252/msb.20177703)
Supplement: Supplementary file 1 — Expanded View Figures PDF [file MSB-13-938-s001.pdf]

Expanded View Figures

Figure EV1. Biological functions enriched in key co-expression clusters of human tissues.

A We examined biological functions (i.e., gene ontology biological process terms) enriched in key co-expression clusters identified in metabolically active tissues, such as skeletal muscle, liver, or adipose subcutaneous or omental tissues (hypergeometric test $P < 0.0001$). We found that those tissues were enriched in different biological functions and fatty acid metabolic process was enriched in liver and adipose subcutaneous tissue.

B We examined "fatty acid metabolic process" term among key co-expression clusters of 46 human tissues and found that liver and adipose subcutaneous tissues were most significantly enriched to this term in their key co-expression clusters.

C We showed all biological functions enriched in 46 human tissues (hypergeometric test $P < 0.0001$). The list of the detailed biological functions can be found in Dataset EV3.

© 2017 The Authors

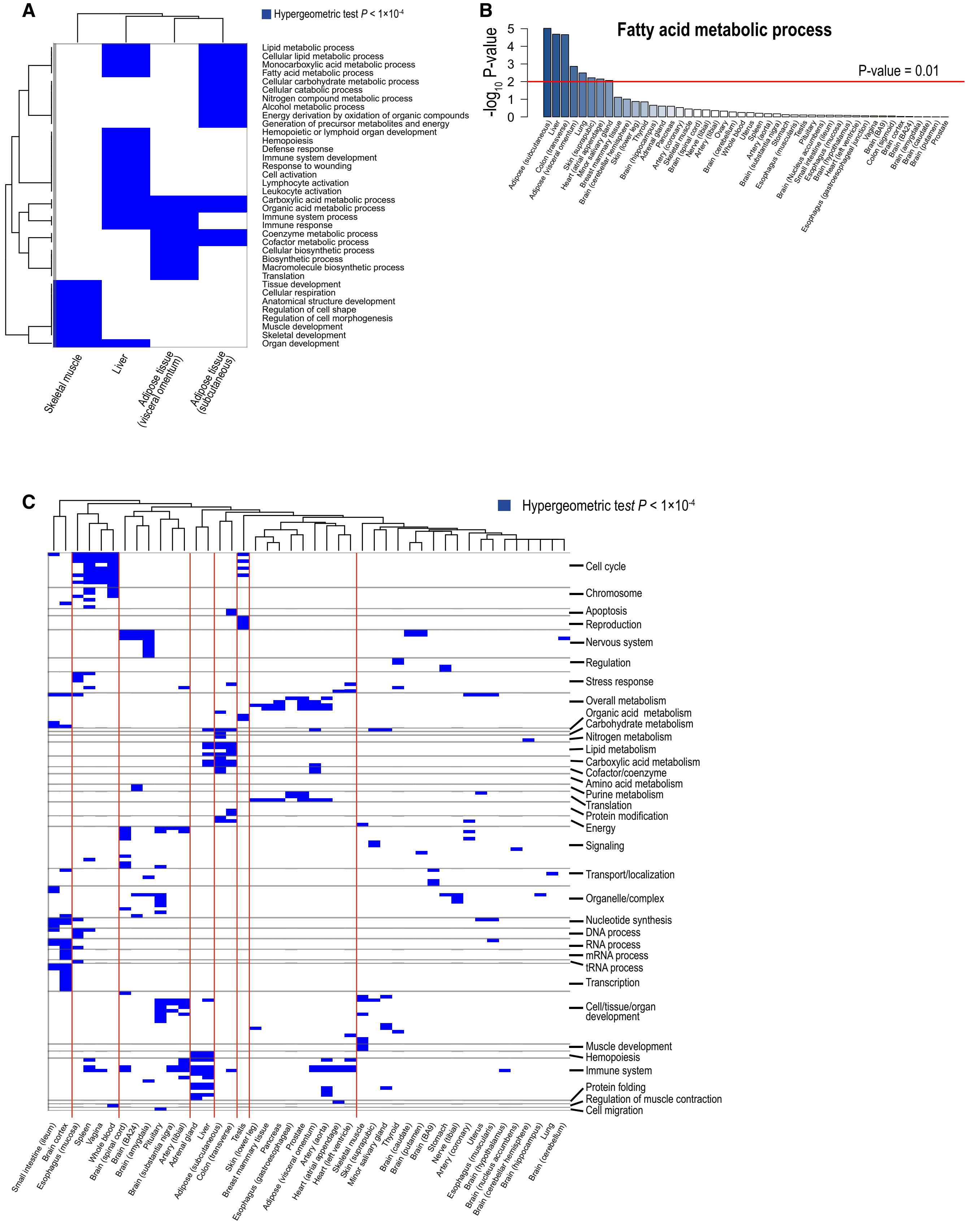
A
Hypergeometric test P < 1×10-4
Lipid metabolic process
Cellular lipid metabolic process
Monocarboxylic acid metabolic process
Fatty acid metabolic process
Cellular carbohydrate metabolic process
Cellular catabolic process
Nitrogen compound metabolic process
Alcohol metabolic process
Energy derivation by oxidation of organic compounds
Generation of precursor metabolites and energy
Hemopoietic or lymphoid organ development
Hemopoiesis
Defense response
Immune system development
Response to wounding
Cell activation
Lymphocyte activation
Leukocyte activation
Carboxylic acid metabolic process
Organic acid metabolic process
Immune system process
Immune response
Coenzyme metabolic process
Cofactor metabolic process
Cellular biosynthetic process
Biosynthetic process
Macromolecule biosynthetic process
Translation
Tissue development
Cellular respiration
Anatomical structure development
Regulation of cell shape
Regulation of cell morphogenesis
Muscle development
Skeletal development
Organ development
Skeletal muscle
Liver
Adipose tissue (visceral omentum)
Adipose tissue (subcutaneous)
B
Fatty acid metabolic process
-log10 P-value
P-value = 0.01
C
Hypergeometric test P < 1×10-4
Cell cycle
Chromosome
Apoptosis
Reproduction
Nervous system
Regulation
Stress response
Overall metabolism
Organic acid metabolism
Carbohydrate metabolism
Nitrogen metabolism
Lipid metabolism
Carboxylic acid metabolism
Cofactor/coenzyme
Amino acid metabolism
Purine metabolism
Translation
Protein modification
Energy
Signaling
Transport/localization
Organelle/complex
Nucleotide synthesis
DNA process
RNA process
mRNA process
tRNA process
Transcription
Cell/tissue/organ development
Muscle development
Hemopoiesis
Immune system
Protein folding
Regulation of muscle contraction
Cell migration

Figure EV1.

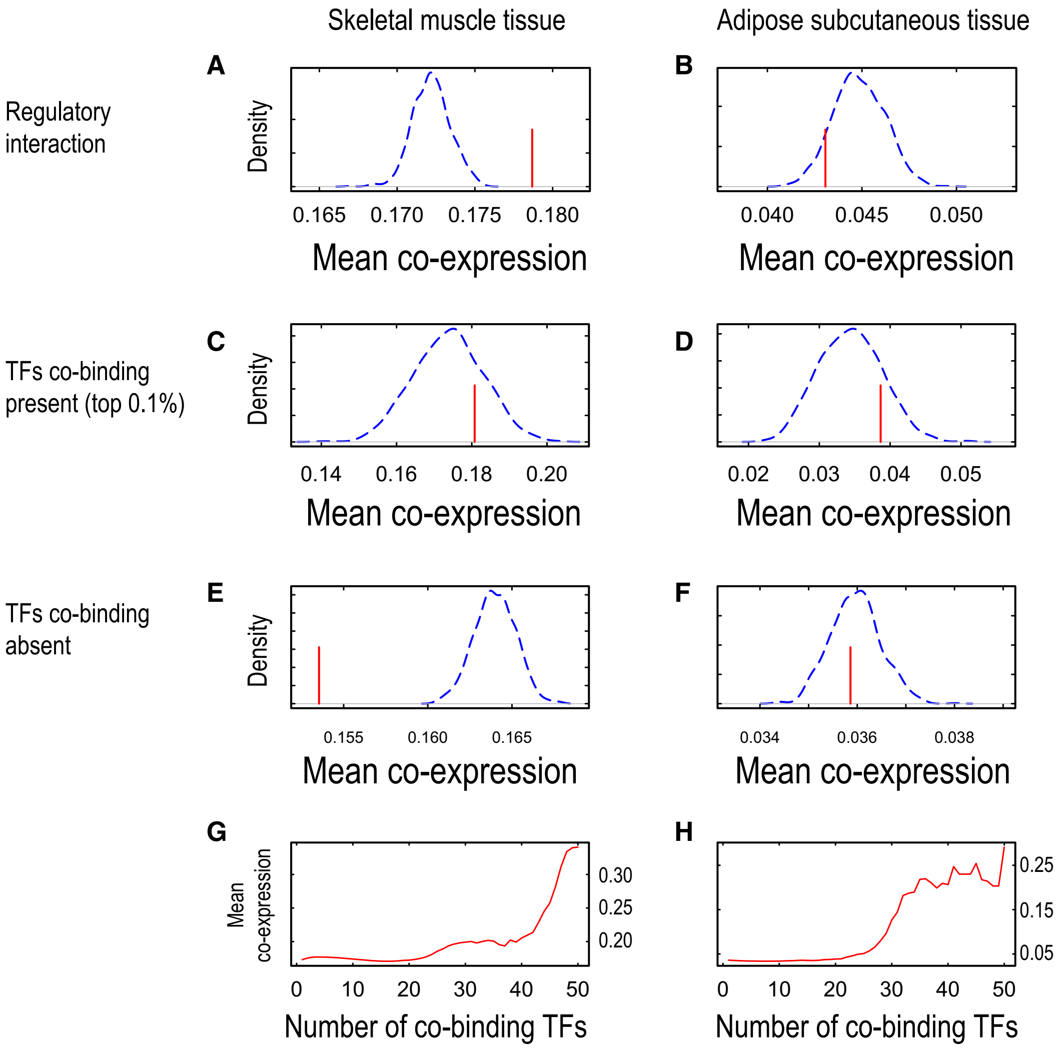

Figure EV2. Co-expressions of regulatory interactions and their co-regulated gene pairs in skeletal muscle and adipose tissue.

A–H We examined mean co-expressions (red lines) of gene pairs involved in regulatory interactions and compared them with randomly permutated gene pairs by 1,000 times (blue dashed lines) for skeletal muscle tissue and adipose subcutaneous tissue (A and B, respectively). We also examined mean co-expressions of gene pairs that are co-regulated by same transcription factors (TFs) in skeletal muscle tissue and adipose subcutaneous tissue (C and D, respectively). For comparison, we selected gene pairs that do not share TFs binding and examined their mean co-expressions together with randomly permutated gene pairs in skeletal muscle tissue and adipose subcutaneous tissue (E and F, respectively). Finally, we examined mean co-expressions of co-regulated gene pairs according to the number of TFs co-bound in skeletal muscle tissue and adipose subcutaneous tissue (G and H, respectively).

 © 2017 The Authors

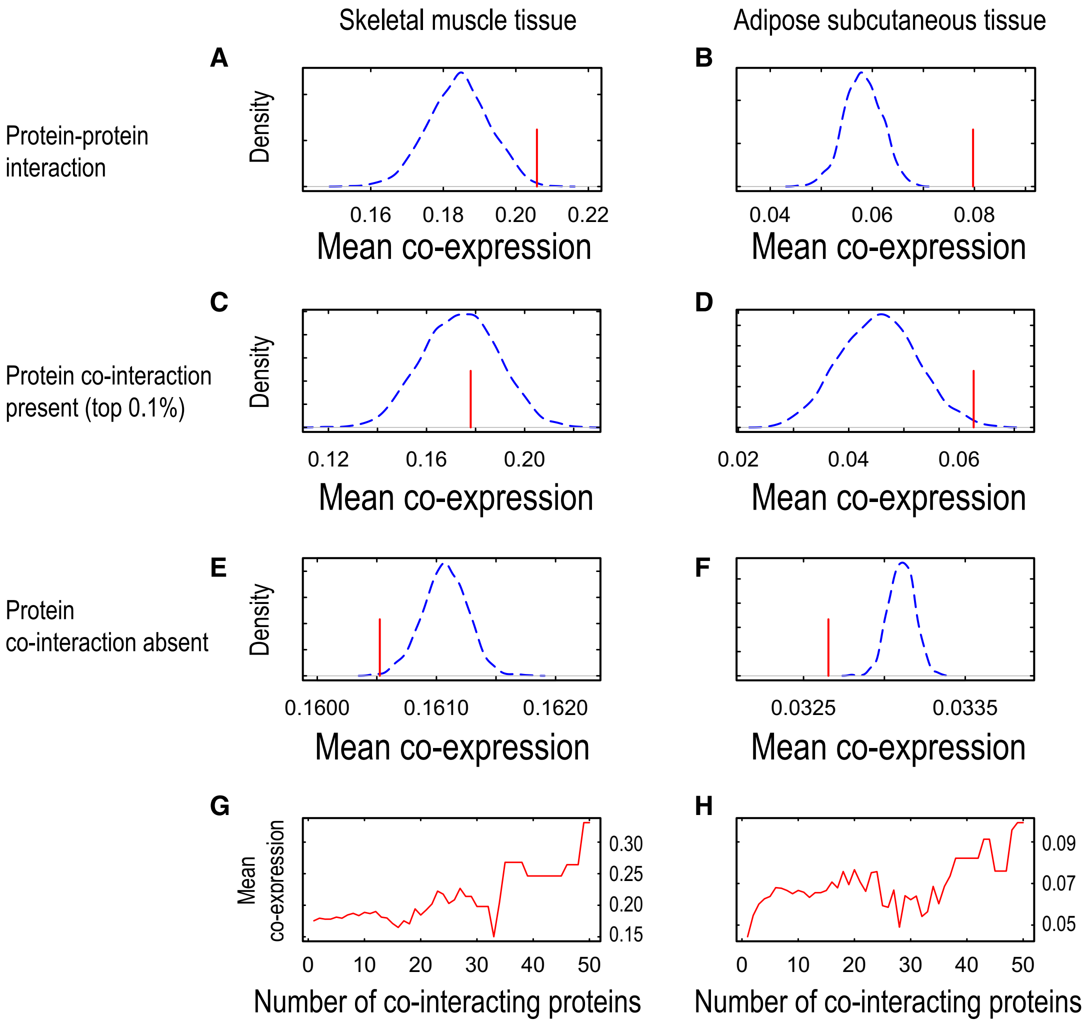

Figure EV3. Co-expressions of protein–protein interactions and their co-interacting gene pairs in skeletal muscle and adipose tissue.

A–H We examined mean co-expressions (red lines) of gene pairs involved in protein–protein interactions and compared them with randomly permutated gene pairs by 1,000 times (blue dashed lines) for skeletal muscle tissue and adipose subcutaneous tissue (A and B, respectively). We also examined mean co-expressions of gene pairs that co-interacted with same proteins in skeletal muscle tissue and adipose subcutaneous tissue (C and D, respectively). For comparison, we selected gene pairs that do not interact with same proteins and examined their mean co-expressions together with randomly permutated gene pairs in skeletal muscle tissue and adipose subcutaneous tissue (E and F, respectively). Finally, we examined mean co-expressions of co-interacting gene pairs according to the number of proteins co-interacting in skeletal muscle tissue and adipose subcutaneous tissue (G and H, respectively).

© 2017 The Authors
